# X-inactivation normalizes *O*-GlcNAc transferase levels and generates an *O*-GlcNAc-depleted Barr body

**DOI:** 10.3389/fgene.2014.00256

**Published:** 2014-08-04

**Authors:** Stéphanie Olivier-Van Stichelen, John A. Hanover

**Affiliations:** Laboratory of Cellular and Molecular Biology, National Institute of Diabetes and Digestive and Kidney Diseases, National Institute of HealthBethesda, MD, USA

**Keywords:** O-GlcNAcylation, Barr body, X-chromosome inactivation, O-GlcNAc transferase, gene silencing

## Abstract

*O*-GlcNAc Transferase (OGT) catalyzes protein *O*-GlcNAcylation, an abundant and dynamic nuclear and cytosolic modification linked to epigenetic regulation of gene expression. The steady-state levels of *O*-GlcNAc are influenced by extracellular glucose concentrations suggesting that *O*-GlcNAcylation may serve as a metabolic sensor. Intriguingly, human *OGT* is located on the X-chromosome (Xq13) close to the X-inactivation center (XIC), suggesting that *OGT* levels may be controlled by dosage compensation. In human female cells, dosage compensation is accomplished by X-inactivation. Long noncoding RNAs and polycomb repression act together to produce an inactive X chromosome, or Barr body. Given that OGT has an established role in polycomb repression, it is uniquely poised to auto-regulate its own expression through X-inactivation. In this study, we examined *OGT* expression in male, female and triple-X female human fibroblasts, which differ in the number of inactive X chromosomes (Xi). We demonstrate that *OGT* is subjected to random X-inactivation in normal female and triple X cells to regulate *OGT* RNA levels. In addition, we used chromatin isolation by RNA purification (ChIRP) and immunolocalization to examine *O*-GlcNAc levels in the Xi/Barr body. Despite the established role of *O*-GlcNAc in polycomb repression, OGT and target proteins bearing *O*-GlcNAc are largely depleted from the highly condensed Barr body. Thus, while *O*-GlcNAc is abundantly present elsewhere in the nucleus, its absence from the Barr body suggests that the transcriptional quiescence of the Xi does not require OGT or *O*-GlcNAc.

## Introduction

*O*-GlcNAc is an abundant and dynamic modification found on approximately 4000 nuclear and cytoplasmic proteins (Ma and Hart, 2014). This post-translational modification (PTM) is added to serine and threonine moieties by *O*-GlcNAc transferase (OGT) and affects various cellular functions including protein stability, transcriptional activity and intracellular localization. OGT utilizes the end product of the hexosamine biosynthetic pathway, UDP-GlcNAc to transfer a single N-acetylglucosamine onto proteins. With 2–3% of intracellular glucose contributing to the hexosamine biosynthetic pathway, OGT is able to sense cellular glucose intake (Marshall et al., 1991) and coordinates an appropriate cellular response by modifying transcription factors, kinases, nuclear pore proteins and RNA Polymerase II. This modification is reversible as a second enzyme, *O*-GlcNAcase (OGA), removes the GlcNAc residue. Through dynamic *O*-GlcNAc cycling, OGT and OGA transform this single PTM into a metabolic sensor (Hanover et al., 2010). After discovery of the *O*-GlcNAc modification (Torres and Hart, 1984), characterization of the enzymes (Braidman et al., 1974; Haltiwanger et al., 1992), and identification of the cDNA encoding OGT (Kreppel et al., 1997; Lubas et al., 1997) the gene was localized to the X-chromosome in mice (XqD) (Shafi et al., 2000) and humans (Xq13) (Hanover, 2001). Knowing that *OGT* is an X-linked gene raises the question of how males (XY) and females (XX) might differentially regulate their OGT levels.

Dosage compensation is the process that equalizes female and male sexual chromosome transcription. In mammals, dosage compensation is accomplished by inactivation of one X chromosome in female cells through a process termed X-inactivation. This mechanism was discovered in 1961 and is now well characterized (Lyon, 1961). Although the majority of X-inactivation studies have been performed using mice, the process is generally accepted to be similar for humans. During the early stages of embryogenesis, the inner cell mass of the embryo has two active X-chromosomes (Patrat et al., 2009) and undergoes X-inactivation by first “counting” to define how many chromosomes need to be inactivated (Gartler and Riggs, 1983). Next, all X-chromosomes but one are chosen for inactivation in a random fashion (Beutler et al., 1962). X-inactivation continues with the synthesis of the long non-coding RNA (lncRNA) *XIST* by the X-inactivation Center (XIC) of the future inactivated chromosome (Brown et al., 1991; Clemson et al., 1996). *XIST* RNA spreads along the chromosome creating a landing platform for recruitment of Polycomb Group (PcG) Repressive complexes (PRC1 and PRC2) (Plath et al., 2003; De Napoles et al., 2004) that deposit repressive histone modifications. Finally, the silencing is maintained by gene promoter DNA methylation (Blewitt et al., 2008). During this process, the inactivated X (Xi) is relocalized to a special nuclear structure called the Barr body (Barr and Bertam, 1949) characterized by high levels of silencing DNA marks and heterochromatin.

X-inactivation is required for female development as deletion of the mouse *X*ist locus, and thus the subsequent cascade of silencing, is lethal for female embryos (Marahrens et al., 1997). Additionally, presence of an Xi is also necessary for normal female development as Turner syndrome patients (XO) exhibit pathologic changes attributed to the presence of only a single X chromosome (Bakalov et al., 2009). These pathologies are largely attributed to X-linked genes that escape X-inactivation. Thus, a number of genes along the Xi remain transcriptionally active despite their location. While mice have proven to be a good model for many human diseases, studies of XO mice have largely been unable to recapitulate phenotypes of Turner syndrome patients (Disteche, 1999). Whereas around 15% of genes on the Xi escape X-inactivation in humans, only about 3% escape in mice. Additionally, there can be spatiotemporal regulation of Xi that may occur for individual genes (Carrel and Willard, 2005; Yang et al., 2010). Therefore, it is important to understand the X-inactivation status of individual genes in human tissues in order to understand the impact of gene dosage on human health and disease.

Intriguingly, it has been demonstrated that the timing of silencing of X-linked genes corresponds to their proximity to the XIC. Genes located close to the XIC are silenced earlier in development than those farther away. Possibly due to its close proximity to the XIC (Shafi et al., 2000), *Ogt* exhibits monoallelic expression in differentiated mouse embryonic stem cells, a developmental time point where most X-linked genes are still biallelically expressed (Lin et al., 2007). Even though the X-inactivation status of *OGT* has not been investigated in humans, indirect evidence suggests that *OGT* silencing is required in humans for normal health. It has been reported that DNA demethylation associated with lupus, an autoimmune disease predominantly affecting females, triggers or worsens disease development (Hewagama et al., 2013). The authors reported that lupus flares correlated with a hypomethylated *OGT* promoter and an increase in OGT levels (Hewagama et al., 2013).

Several studies have demonstrated an association between OGT and the PcG proteins, a group of proteins integral to the X-inactivation mechanism (Plath et al., 2003; De Napoles et al., 2004), suggesting that OGT could be regulating its own expression. The homeotic gene Super sex combs (*Sxc*) in *Drosophila* encodes OGT and is required for PcG group function (Sinclair et al., 2009). Additionally, *O*-GlcNAc is abundant at PcG-binding sites on polytene chromosomes (Gambetta et al., 2009). Finally, PRC2 is tightly linked to OGT levels in mouse embryonic stem cells (Myers et al., 2011). Therefore, OGT might act through polycomb to influence its own X-inactivation, thus creating a feedback regulatory loop. In addition, *O*-GlcNAc may play a role in the transcriptional silencing associated with the Barr body, hosting the Xi. Here, we examined *OGT* expression in male, female and triple-X female human fibroblasts, all of which have different X-inactivation statuses. We demonstrate that *OGT* is subjected to X-inactivation in female and triple-X cells to normalize OGT levels. We were surprised to find that the Barr body, containing the Xi, is substantially depleted of both *O*-GlcNAc and OGT relative to other intranuclear locations. This suggests that high levels of *O*-GlcNAc are not associated with the transcriptionally silenced Xi in the Barr body.

## Materials and methods

### Cell culture

GM00254 (47, XXX), GM00468 (46, XY) and GM6111 (46,XX) cell lines were purchased from the NIGMS Human Genetic Mutant Cell Repository and grown according to the recommended conditions.

### Drugs and RNAi

5-azacytidine was used at 10 μM (Sigma Aldrich®), incubated overnight in optiMEM and then for 2 days in classic cell culture medium. Thiamet-G was used at 100 nM, azaserine at 5 μM, both for 2 h in fresh medium. *XIST* RNAi was purchased from Origen® and used according to the recommended protocol. Briefly, cells were transfected either with *XIST* or scrambled, control RNAi using lipofectamine RNAimax (Invitrogen®). Cells were incubated overnight in optiMEM medium and then for 2 days in classic culture medium.

### RT-PCR and quantitative RT-PCR (qRT-PCR)

Cells were washed twice with PBS and then trypsinized for 5 min at room temperature. Cells were then washed twice with PBS and total RNAs were purified with RNeasy minikit (Qiagen®), including a DNAse step, and recovered in 20 μL of PCR-grade water. RNA concentration was measured using the nanodrop dosage spectrophotometer, and quality was checked by running RNA on an agarose gel and analyzing the integrity of ribosomal RNA (28S and 18S). Four micro litter of qScript cDNA Supermix (Quanta bioscience) was added to 500 μg of RNA extract and run on a thermocycler to provide cDNA using the following program: 25°C/5 min; 42°C/30 min; 85°C/5 min: 10°C/8. Total cDNA was then quantified using nanodrop spectrophotometer.

For classic RT-PCR, 1 μg of cDNA was mixed with 1 μL of ampliTaq DNA polymerase (5U/μL, Applied biosystems®), 0.5 μL of each primer (100 μM), 25 μL of failsafe PCR 2x premix (Epicenter®) (in 50 μL PCR grade water). Amplification was performed on a thermocycler using the following program: 95°C/3 min; 95°C/1 min; 85°C/5 min: (Primer hybridization temperature)/1 min; 72°C/1 min/kb (repeat three last steps 40×); 72°C/2 min; 4°C/8. Finally, samples were run on 2% agarose E-gel (Invitrogen).

For qRT-PCR, 1 μg of cDNA was mixed with 10 μL of fast SYBR Green master mix (Applied Biosystems®) and 0.5 μL of each primer (100 μM) (in 20 μL PCR-grade water) and amplified on AB 7900HT fast real time PCR system (Applied biosystems). Each experiment was performed in triplicate, analyzed by calculating the 2^−ΔΔCT^ (Livak and Schmittgen, 2001) relative to actin expression and significance was calculated with the one-way ANOVA test (GraphPad Prism v.6).

### Primers

Actin Ex-Ex (Exon-Exon junction): For (5′→3′) CCTTCCTTCCTGGGCATGGAGT CC; Rev (5′→3′) GGAGGAGCAATGATCTTGATCTTCC. G6PD intron: For (5′→3′): GGCATCAGCAAGACACTCTCTCC; Rev (5′→3′) ACCCCATAGCCCACAGGTAT GC. *OGT* intron: For (5′→3′) AGTTGTTATCTTTTGTATTACA; Rev (5′→3′) AATTTGTTCAAGGAAAAGTTTACTTA. *OGT* Ex-Ex: For (5′→3′) GGTGACTATGC CAGGAGAGACTCTTGC; Rev (5′→3′) CGAACTTTCTTCAGG TATTCTAGATC. *XIST* Ex-Ex: For (5′→3′) GGCTCCTCTTGGACATTCTGAGC; Rev (5′→3′) CTTCTGAGGAAGATCTTTTTCTCC.

### RNA fluorescence *in situ* hybridization (RNA-FISH)

Cells were cultured on 2-chamber-glass-slides (Electron Microscopy Sciences) for one day. One hour before fixation, fresh medium was added to the chamber-glass-slides. Slides were then treated according the multiplex fluoresent assay RNAscope® (Advanced Cell Diagnostics) protocol. Briefly, cells were washed twice with PBS and then incubated for 10 min at RT with 10% Neutral Buffered Formalin (NBF) (10% Formaldehyde, 30 mM NaH_2_PO_4_, 45 mM Na_2_HPO_4_). After two PBS washes, cells were dehydrated successively in 50, 70, and 100% ethanol for 1 min each. Finally, cells were incubated in fresh 100% ethanol for 10 min at RT. Cells were then rehydrated successively in 70% ethanol, 100% ethanol and finally in PBS for 1min each at RT. Finally, cells were incubated in fresh PBS for 10 min at RT. Cells were then treated with pretreat3 solution (RNAscope® pretreatment Kit –FL FF, Advanced Cell diagnostics), containing protease for 10 min at RT. After two PBS washes, cells were incubated with Human XIST lncRNA/OGT pre-mRNA mixed probe or Human 2-plex-postive control probe (containing PPIB and RNA polymerase II mRNA probes) or Human 2-plex-negative control probe (containing two bacterial mRNA probes) for 3 h at 40°C. Probe detection was performed following the detection kit protocol provided by Advanced Cell Diagnostics (RNAscope® Detection Kit –FL). Finally, cells were mounted in DAPI/fluoromount G (Electron Microscopy Sciences). After drying, slides were observed under a confocal microscope (Zeiss LSM700).

### Immunofluorescence

Cells were cultured on 2-chamber-glass-slides (Electron Microscopy Sciences) for two days. Cells were then washed twice with PBS and fixed in freshly made 1% paraformaldehyde/PBS for 15 min at room temperature. After three PBS washes (5 min each), cells were permeabilized at 4°C for 15 min in 1% triton-X100/PBS. After three PBS washes (5 min each), slides were saturated in a 1% BSA/PBS solution for 15 min at room temperature and incubated with antibodies diluted at 1:200 in 1% BSA/PBS for 1h at room temperature. After three PBS washes (5 min each), cells were incubated with alexa-fluor 488 anti-mouse IgG or 568 anti-rabbit IgG secondary antibodies (Life Technologies) diluted at 1:500 in 1% BSA/PBS for 30 min at room temperature and in the dark. Finally, cells were washed in PBS three times and mounted in DAPI/fluoromount G (Electron Microscopy Sciences). After drying, slides were observed under a confocal microscope (Zeiss LSM700).

### Chromatin isolation by RNA purification (CHIRP)

ChIRP was carried out as previously described (Chu et al., 2011). Briefly, 22 probes were designed to recognize the human *XIST* RNA fragment from 10.5 to 13.5 kb (using www.singlemoleculefish.com). Each probe was 20 bp in length and separated from the next by at least 80 bp (1 probe/100 bp). Probes were numbered following the sequence from 1 to 22 and then separated in 2 pools (odd and even), which were used as internal controls for each other. Specific RNA and DNA content should be found in both pools. Anti-sense DNA probes were labeled with a 3′-end BiotinTEG.

Cells were washed twice with PBS and then trypsinized at room temperature (RT) for 5 min. Cells were again washed twice with PBS, cross-linked at RT for 10 min in 400 μL of 1% glutaraldehyde/PBS and then quenched by 400 μL cold glycine at 250 mM (final concentration 125 mM) for 5 min at 4°C. After centrifugation at 4°C for 5 min at 2500 rcf, and two PBS-washes, pellets were resuspended in lysis buffer (10× mass pellet in grams) [*50 mM Tris-Hcl pH 7, 10 mM EDTA pH8, 1% SDS, protease inhibitors (Roche)*] with freshly added 1mM of PMSF (Fluka) and Superase-in (Ambion). Lysate was sonicated (water bath-sonicator) at 4°C for at least 30 min (30 s ON, 45 s OFF, output 7) or until the sample was no longer turbid. After centrifugation at 20,000 rcf for 10 min, supernatant was kept for ChIRP. After putting aside samples for DNA and RNA input, lysates were diluted in 2 volumes of hybridization buffer (*50 mM Tris-Hcl pH 7, 750 mM NaCl, 10 mM EDTA pH8, 1% SDS, 15% forfamide, protease inhibitors)* supplemented freshly by 1 mM of PMSF and Superase-in, and separated into two tubes for the two pools of probes (Odd and Even). Probes were added at 100 pmol probe/mL of initial lysate (before hybridization buffer) and incubated for 4 h at 37°C. After washing the C-1 magnetic beads (Invitrogen) twice with lysis buffer, beads were added into the lysate for 1h at 37°C (100 μL beads/mL initial lysate). Beads were then washed five times by shaking for 5 min at 37°C in pre-warmed washing buffer (2× Saline Sodium Citrate (SSC) 0.5*% SDS, proteases inhibitors)* supplemented by fresh 1 mM PMSF and Superase-in. After the last wash, beads were resuspended in 1 mL of washing buffer and separated for DNA and RNA purification as described in ChIP.

### Chromatin precipitation (ChiP)

Cells were washed twice with PBS and then trypsinized at RT for 5 min. Cells were then washed twice with PBS, cross-linked at 4°C for 10 min in 400 μL 1% paraformaldehyde/PBS and then quenched by 400 μL cold glycine at 250 mM (final concentration 125 mM) for 5 min at 4°C. After centrifugation at 4°C for 5 min at 2500 rcf, pellets were resuspended in 250 μL of lysis buffer [*50 mM HEPES-KOH pH 7.5, 140 mM NaCl, 5 mM EDTA pH8, 1% Triton-X100, 0.5% NP-40, 0.5mM DTT, 1% SDS, protease inhibitors (Roche)*] for 10 min on ice with occasional shaking. Chromatin fragmentation was done by two sonication cycles (water bath-sonicator) of 10 min (output 7) with 1 min break on ice between. Lysate was cleared by centrifugation at 20,000 × g for 10 min and supernatant was stored at −80°C if needed. Lysate was incubated at 65°C for 1.5 h to reverse the cross-linking and kept for total mRNA and total protein extraction. Forty micro litter of protein G magnetic beads (Cell Signaling®) were washed twice with SDS free RIPA buffer *(50 mM Tris-HCl pH8, 150 mM NaCl, 2 mM EDTA pH8, 1% NP-40, 0.5% sodium deoxycholate, protease inhibitors)* and incubated for 1 h at 4°C in SDS-free RIPA buffer with 0.1 μg/μL BSA (New England biolabs) and 75 ng/μL ssDNA (Salmon sperm DNA, Invitrogen). Finally, beads were washed with SDS free RIPA buffer and resuspended in SDS free RIPA buffer with 0.1 μg/μL BSA and 75 ng/μL ssDNA. Remaining lysate was also diluted in the previous solution up to 1 mL with 10 μL of ChIP-grade anti-mH2A1 antibody (ab37264, Abcam) for 1 h at 4°C. Prepared beads were then added in tubes and incubated overnight at 4°C. Beads were captured on magnetic rack and wash three times at 4°C with RIPA/SDS *(20 mM Tris-HCl pH8, 150 mM NaCl, 2 mM EDTA pH8, 1% triton-X100, 0.1% SDS)* and then three times at 4°C with high stringency RIPA buffer *(20 mM Tris-HCl pH7.5, 500 mM NaCl, 2 mM EDTA pH8, 1% triton-X100, 0.1% SDS)*. Complexes were eluted from beads by adding 60 μL of elution buffer *(100 mM NaHCO_3_, 1% SDS)* and incubating at 30°C for 20 min. Beads were removed by capture on the magnetic rack and supernatants were incubated at 65°C for 1.5 h to reverse the cross-linking. Samples were then divided in three to analyze the DNA, RNA and protein contents.

### ChiRP and ChiP samples analysis

For protein analysis, 4× Laemmli sample buffer was added to both input and immunoprecipited samples and boiled at 100°C for 10 min before loading on a Nupage Novex 10% Bis-Tris gel (Life Technologies®). Proteins were then transferred to a nitrocellulose membrane (Life Technologies®). Membranes were first incubated for 1 h at room temperature with the Odyssey blocking buffer (LI-COR Biosciences®) and then incubated overnight with primary antibodies diluted in Odyssey blocking buffer at 4°C. After three washes of 10 min with PBS-tween *(0.05% Tween-20)*, membranes were incubated with secondary anti-mouse IRDye 680LT or anti-rabbit IRDye 800CW antibodies (LI-COR Biosciences) diluted at 1:10,000 in PBS-tween for 1h at room temperature in the dark. After, three final washes of 10 min with PBS-tween membranes were imaged using the Odyssey Infrared Imaging System (LI-COR Biosciences, Lincoln, NE) according to the manufacturer's instructions.

For DNA purification, samples (input and purified) were first incubated with RNAse A (1 mg/mL) (Invitrogen®) at 37°C for 2 h in DNA elution buffer *(50 mM NaHCO3, 1% SDS)*. Supernatant was then separated from beads, if necessary, and each sample was incubated with 15 μL of proteinase K at 50°C for 45 min. Samples were then boiled at 95°C for 10 min and DNA fragments were purified with PCR purification kit (Quiagen). PCR was performed as described previously for cDNA.

For RNA purification, 10 μL of RNA samples (input and purified) were first treated with proteinase K (5 μL) in proteinase K buffer (85 μL) *(10 mM Tris-HCl pH 7, 100 mM NaCl, 1 mM EDTA, 1% SDS)* for 45 min at 50°C. Samples were boiled at 95°C for 10 min and RNA was purified with RNeasy minikit (Quiagen) and analysis was done as previously described.

### Antibodies

For western blot experiments, ChIP grade mH2A1 (Abcam, ab37264), GAPDH (Abcam, ab9484), H2AK119Ub (Millipore, #AB10029) and *O*-GlcNAc (RL2) (Thermoscientific, MA1-072) antibodies were used at a dilution of 1:1000. For immunofluorescence experiments, *O*-GlcNAc (RL2) (Thermoscientific, MA1-072), OGT (TI14) (Sigma Aldrich, O6014), RNA polymerase II (phospho S2) (Abcam, ab5095 and ab5408), RNA polymerase II (phospho S2/5) (Cell signaling, #4735), Actin (Abcam, ab1801) and H3K27Me3 (Millipore, #07-449) antibodies were used at a dilution of 1:200, mH2A1 (Cell signaling, #4827) and pan RNA polymerase II (RBP8) (Abcam, ab169924) at a dilution of 1:100.

## Results

### *OGT* is subjected to X-inactivation to maintain *OGT* transcript levels

**Figure 1:**
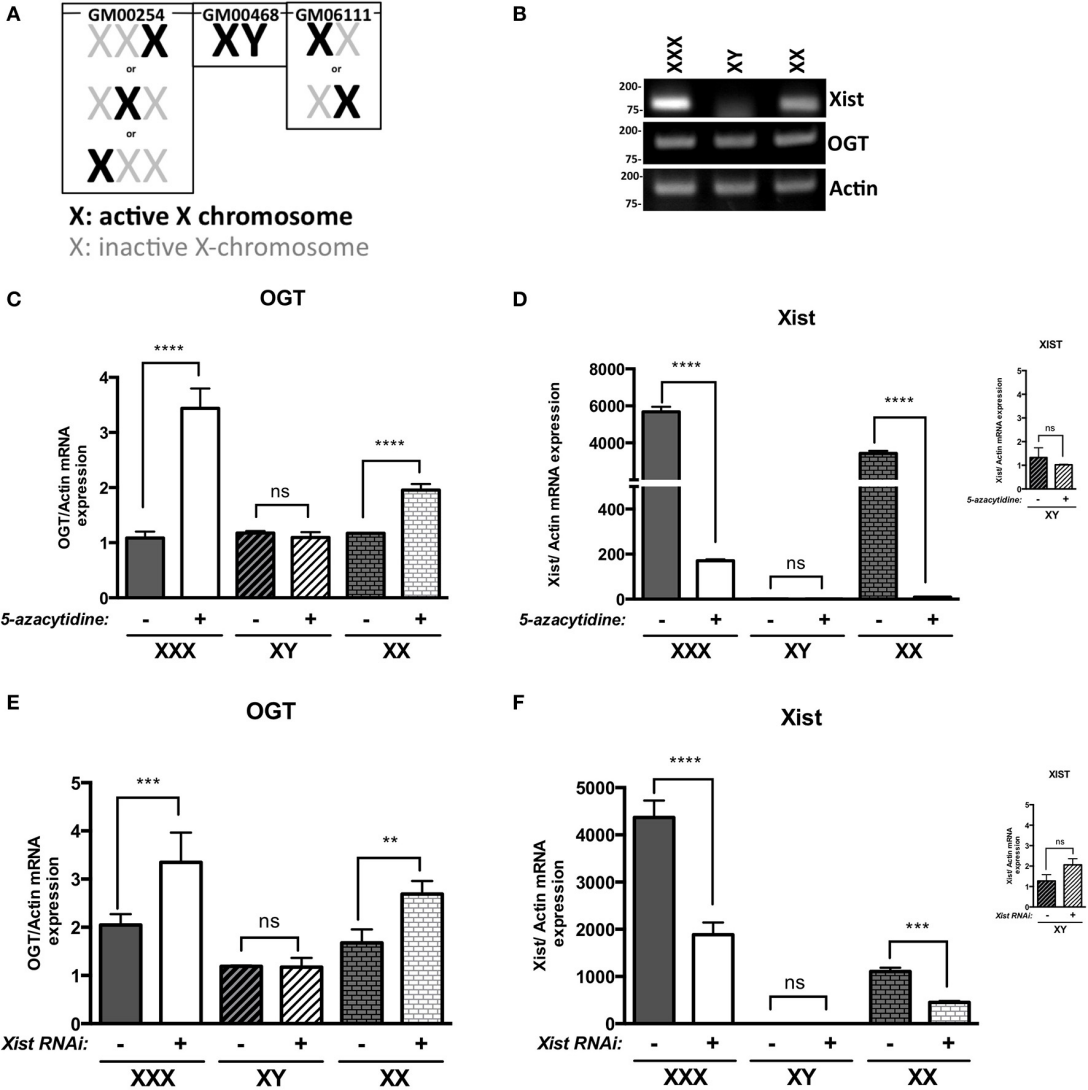
***OGT* is subject to X-inactivation in females**. **(A)** Male (GM00468), Female (GM6111) and triple-X (GM00254) human fibroblasts show different X-inactivation profiles: no Xi in male, one Xi in female and 2 Xi in triple-X female cells. **(B)** *XIST* mRNA expression, analyzed by RT-PCR, is increased with Xi status. *OGT* expression remains the same in the different cell lines. **(C)** 5-azacytidine, a DNA methylation inhibitor, increases *OGT* mRNA expression according to the number of Xi, analyzed by qRT-PCR and performed in triplicate. **(D)** *XIST* mRNA expression is decreased in the presence of 5-azacytidine, showing the decrease in components required for X-inactivation. **(E)** *XIST* RNAi treatment also increases *OGT* mRNA expression according to the number of Xi in these human fibroblasts as detected by qRT-PCR performed in triplicate. **(F)** Efficiency of *XIST* RNAi was confirmed by a decrease in *XIST* mRNA expression by qRT-PCR. NS: *P* > 0.1; ^*^0.1 > *P* > 0.01; ^**^0.01 > *P* > 0.001; ^***^0.001 > *P* > 0.0001; ^****^0.0001 > P.

We first investigated *OGT* expression in different human fibroblast cell lines: GM00254 (XXX, triple-X female), GM00468 (XY-male) and GM06111 (XX-female). In these XX and XXX cell lines, X-inactivation has occurred and follows the normal X-inactivation n-1 rule (Lyon, 1996; Lee and Jaenisch, 1997): only one X-chromosome is activated per cell, the others are inactivated (Figure 1A). X-inactivation status was determined by the expression of *XIST* lncRNA and, as expected, male (XY) had lower levels of *XIST* lncRNA relative to female (XX) cells that have undergone X-inactivation (Figure 1B). Moreover, *XIST* levels increased further in triple-X female (XXX), which have two inactivated X-chromosomes. Levels of *OGT* mRNA did not differ in male, female or triple-X cells despite the increased copy number of *OGT* in these cell lines (Figure 1B). Next, male, female and triple-X female human fibroblasts were subjected to 5-azacytidine treatment. This cytidine analog blocks DNA methyltransferase thereby decreasing DNA methylation, which is partly responsible for Xi-silencing. Without the addition of 5-azacytidine (−), the different cell lines showed the same pattern observed by classic RT-PCR (Figure 1C). 5-azacytidine treatment (+) did not affect *OGT* expression in male cells (XY), consistent with the absence of X-inactivation. In contrast, female cells and triple-X cells' *OGT* expression increased by ~ 2 and 3 fold relative to *OGT* expression before 5-azacytidine treatment, respectively. These data are consistent with the presence of two and three copies of OGT alleles in female and triple-X female cells. Interestingly, the relative expression of *XIST* is also greatly reduced after 5-azacytidine treatment in female and triple-X female cells (Figure 1D).

Because 5-azacytidine affects genome-wide DNA methylation and not only X-chromosome silencing, we investigated X-inactivation by using RNA interference against XIST mRNA. Similar to the addition of 5-azacytidine, male cells were not affected by *XIST* RNAi treatment (+) in comparison to control RNAi (−) (Figures 1E,F). Knocking-down *XIST* expression led to significant increases in relative *OGT* expression in female and triple-X cells (Figure 1E). The efficiency of the RNAi transfection was confirmed by a decrease of *XIST* RNA expression (Figure 1F). Taken together, these results suggest that the *OGT* locus is silenced by X-inactivation.

**Figure 2:**
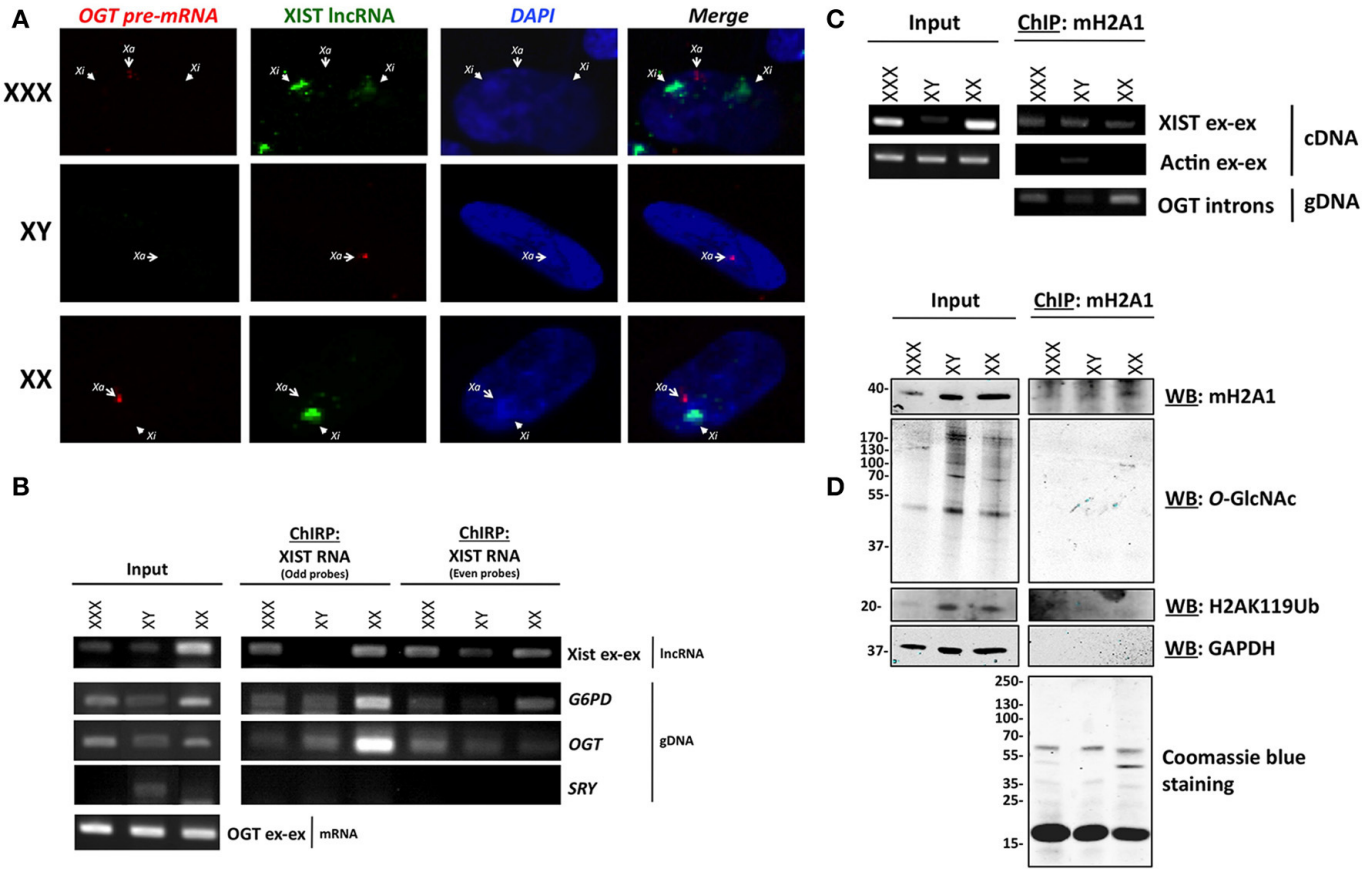
***OGT* is expressed from Xa and extra-*OGT* alleles are part of the X-inactivation complex**. **(A)** RNA-FISH analysis of the *OGT* primary transcript and XIST lncRNA in male, female and triple-X female cells indicates that *OGT* expression does not co-localize with the *Xist* lncRNA. *Xa: active X-chromosome; Xi: inactive X-chromosome*. **(B)** Chromatin isolation by RNA Purification (ChIRP) of *XIST* RNA, a major component of Barr body, shows interaction of the *OGT* locus with *XIST* mRNA. As a control, *G6PD*, another X-linked gene is also found to interact, but not *SRY*, which is on the Y chromosome. **(C)** Chromatin-Immunoprecipitation of mH2A1, another key component of the Barr body demonstrates interactions with the *OGT* gene and *XIST* mRNA. **(D)** Chromatin-Immunoprecipitation of mH2A1 also demonstrates that no *O*-GlcNAcylated proteins are associated with the immuno-precipitated complexes.

We next hypothesized that one allele of *OGT* is transcribed in each cell and that additional *OGT* alleles are associated with major components of the Barr body, such as *XIST* RNA and mH2A1. To determine the transcriptional status of *OGT*, we observed the localization of the *OGT* primary transcript in comparison to *XIST* lncRNA by RNA-FISH. The *OGT* primary transcript was detected using an intronic probe. We observed that *XIST* lncRNA spreads along the inactive X (Xi), and were able to observe 0, 1 or 2 spots in male, female and triple-X female (Figure 2A), respectively. In contrast, *OGT* primary transcript was only synthesized by the active X (Xa), and was synthesized by only one X-chromosome in male female or triple-X female (Figure 2A). As a positive control, *PPIB* [Bos Taurus Peptidylprolyl Isomerase B (cyclophilin B)] and *RNA polymerase II* mRNA were localized throughout the cells (Figure S1A). As a negative control, we were unable to detect bacterial DNA using 2 probes in these human cells (Figure S1A). This experiment confirms that OGT is synthesized only from one chromosome in these cell lines and that additional *OGT* alleles are silenced by the spread of *XIST* lncRNA.

In order to determine if *OGT* is associated with components of the Barr body, we performed Chromatin-Isolation by RNA Purification (ChIRP) of *XIST* lncRNA in male, female, and triple-X female cells. The ChIRP assay pulls down specific RNA via biotinylated probes after cross-linking. The mRNA/DNA contents were then investigated in total or purified extracts for each cell line (Figure 2B). With the two sets of *XIST* RNA probes, serving as a control for each other, *XIST* RNA was pulled-down along with *G6PD* (Glucose-6-phosphate dehydrogenase), a well-known X-inactivated gene (Xq27) (Szabo et al., 1984; Martini et al., 1986). *XIST* was specifically amplified using primers that spanned exon-exon junctions. *G6PD* gene was identified by PCR using intronic primers. As a control, we did not find detect the *SRY* gene, which is on the Y chromosome, except in the male input condition. As expected, we also pulled down the *OGT* gene, using specific intronic primers, with both sets of probes, indicating that the *OGT* locus is associated with *XIST* RNA cells (Figure 2B).

We then performed a Chromatin-Immunoprecipitation (ChIP) followed by RT-PCR or PCR, using an mH2A1antibody, as another representative component of the Barr body (Mermoud et al., 1999). Protein, mRNA and DNA contents were investigated in total or as immunoprecipitated extracts for each cell line (Figures 2C,D). As expected, we found mH2A1 in association with the *XIST* lncRNA as well as *OGT* DNA (Figure 2C). By protein analysis, we showed that ubiquitinylated lysine 119 of histone H2A (H2AK119Ub) co-immunoprecipitated, but not the negative control GAPDH (glyceraldehyde-3-phosphate dehydrogenase) (Figure 2D).

These biochemical experiments confirmed that the *OGT* locus is part of an X-inactivation complex, composed at least of *XIST* lncRNA, mH2A1 and H2AK119Ub (Figures 2C,D). Although we were able to detect other proteins that immunoprecipatated with mH2A1 by coomassie blue staining, we found very few *O*-GlcNAcylated proteins associated with the Barr body purification (Figure 2D). This finding was unexpected given the literature linking *O*-GlcNAc cycling to PcG proteins and Polycomb repression in mammals. Polycomb repression is known to play a key role in X-inactivation leading to extensive compaction to form the highly heterochromatinized Barr body. To better understand our findings, we investigated whether OGT and *O*-GlcNAc targets were present in the Barr body.

### The barr body is enriched for repressive epigenetic marks but depleted of *O*-GlcNAc and OGT

**Figure 3:**
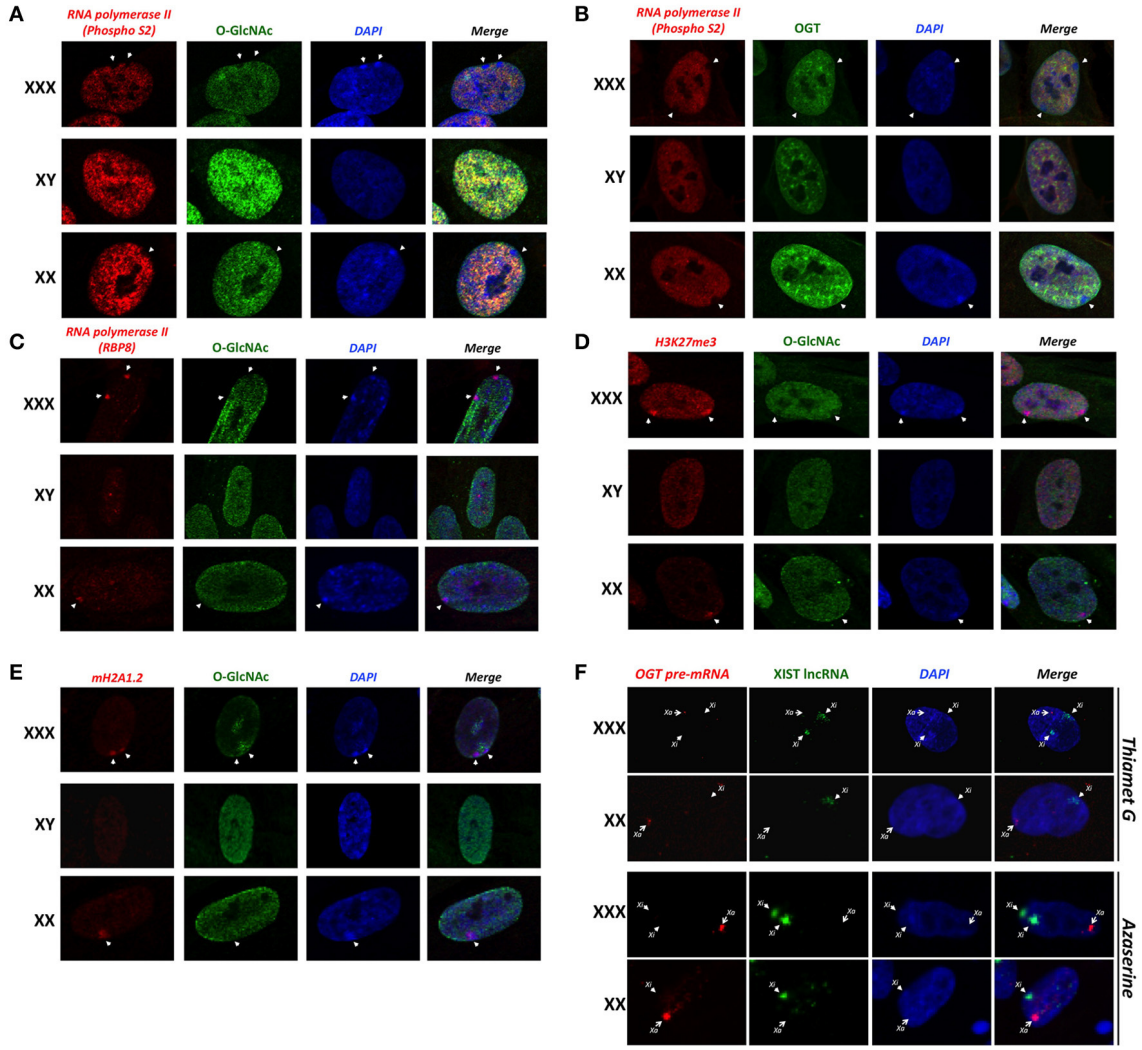
**Barr bodies do not require *O*-GlcNAc cycling. (A)** A strong-DAPI staining signal and/or a lack of elongating RNA polymerase II (PhosphoS2) signal were used to localize Barr bodies (white arrows). *O*-GlcNAc staining is largely excluded from Barr body. **(B)** OGT is also less intense in Barr body. **(C)** Unlike elongating RNA polymerase II (PhosphoS2), unmodified RNA polymerase II co-localized with the Barr body. **(D)** Barr bodies were identified as in this figure. H3K27me3 strongly co-localized with Barr bodies, suggesting a highly silenced region. **(E)** mH2A1 also co-localized with Barr bodies. In both experiments, *O*-GlcNAc staining did not co-localize with these Barr body-specific staining regions. **(F)** RNA-FISH analysis of the *OGT* primary transcript and XIST lncRNA in female and triple-X female, in presence of azaserine of Thiamet G. *Xa: active X-chromosome; Xi: inactive X-chromosome*. Immunofluorescence pictures are representative of triplicate experiments.

Previous studies have shown that *O*-GlcNAc is an abundant modification in interphase nuclei, and found highly concentrated at nuclear pores and subdomains within the nucleus (Hanover, 2001). Similarly, we detected an intranuclear distribution of OGT (Figure 3). We sought to determine whether *O*-GlcNAc might be associated with Barr bodies. The Barr body is known to have a unique chromatin signature maintained by excluding some chromatin modifiers while enriching others (Chadwick and Willard, 2003). Morphological inspection of the Barr body in interphase nuclei has proven to be a useful tool in order to look at numerous chromatin modifications including methylation and acetylation (Chadwick and Willard, 2003). To identify the Barr body, we initially looked for the exclusion of elongating RNA polymerase II (phospho-S2) staining on the periphery of the nucleus. With immunostaining of elongating RNA polymerase II, we identified 0, 1 or 2 Barr bodies in male (XY), female (XX) and triple-X (XXX) human fibroblasts, respectively (Figures 3A,B; white arrows). The identified Barr bodies were also DAPI-rich as it has been reported (Peters et al., 2002), likely due to an increased amount of chromatin compaction of heterochromatin. Surprisingly, the Barr bodies lacked staining of *O*-GlcNAcylation (Figure 3A) and OGT (Figure 3B). In order to demonstrate that the exclusion of *O*-GlcNAcylation was not due to an artifact of a specific antibody, we confirmed the exclusion using another phospho-S RNA polymerase II antibody (phospho S2/5) (Figure S1B). Single secondary antibody staining also confirmed the specificity of observed signals (Figure S1C). However, unphosphorylated RNA polymerase II colocalized with the Barr bodies (Figure 3C), which were identified in this set of experiments by both a lack of *O*-GlcNAc staining and strong DAPI staining. Together, these data show that the Barr bodies exclude only the elongating form of RNA polymerase II. Exclusion of *O*-GlcNAc from the inactive X was confirmed by comparing *O*-GlcNAc staining with H3K27Me3 and mH2A1 staining, both highly enriched in the Barr body (Figures 3E,F).

Because *O*-GlcNAc seems to be largely excluded from the Xi/Barr body, we next hypothesized that modulation of *O*-GlcNAc probably would not likely alter X-inactivation or Barr body formation. We examined the in situ staining of the *OGT* primary transcript in comparison to *XIST* lncRNA by RNA-FISH in presence of Thiamet G or azaserine (Figure 3F). Thiamet G is an OGA inhibitor and increases *O*-GlcNAc levels (Figure S1D). Azaserine inhibits GFAT (Glutamine fructose-6-phosphate amidotransferase), a key enzyme of the HBP, thereby decreasing UDP-GlcNAc levels and *O*-GlcNAcylation (Figure S1D). In both conditions, and in both female and triple-X female, modulating *O*-GlcNAcylation does not effect the localization of the *OGT* primary transcript in relation to *XIST* (Figures 2A, 3F). Taken together, this result suggests that *O*-GlcNAc cycling is not required for X-inactivation or Barr body formation.

## Discussion

**Figure 4:**
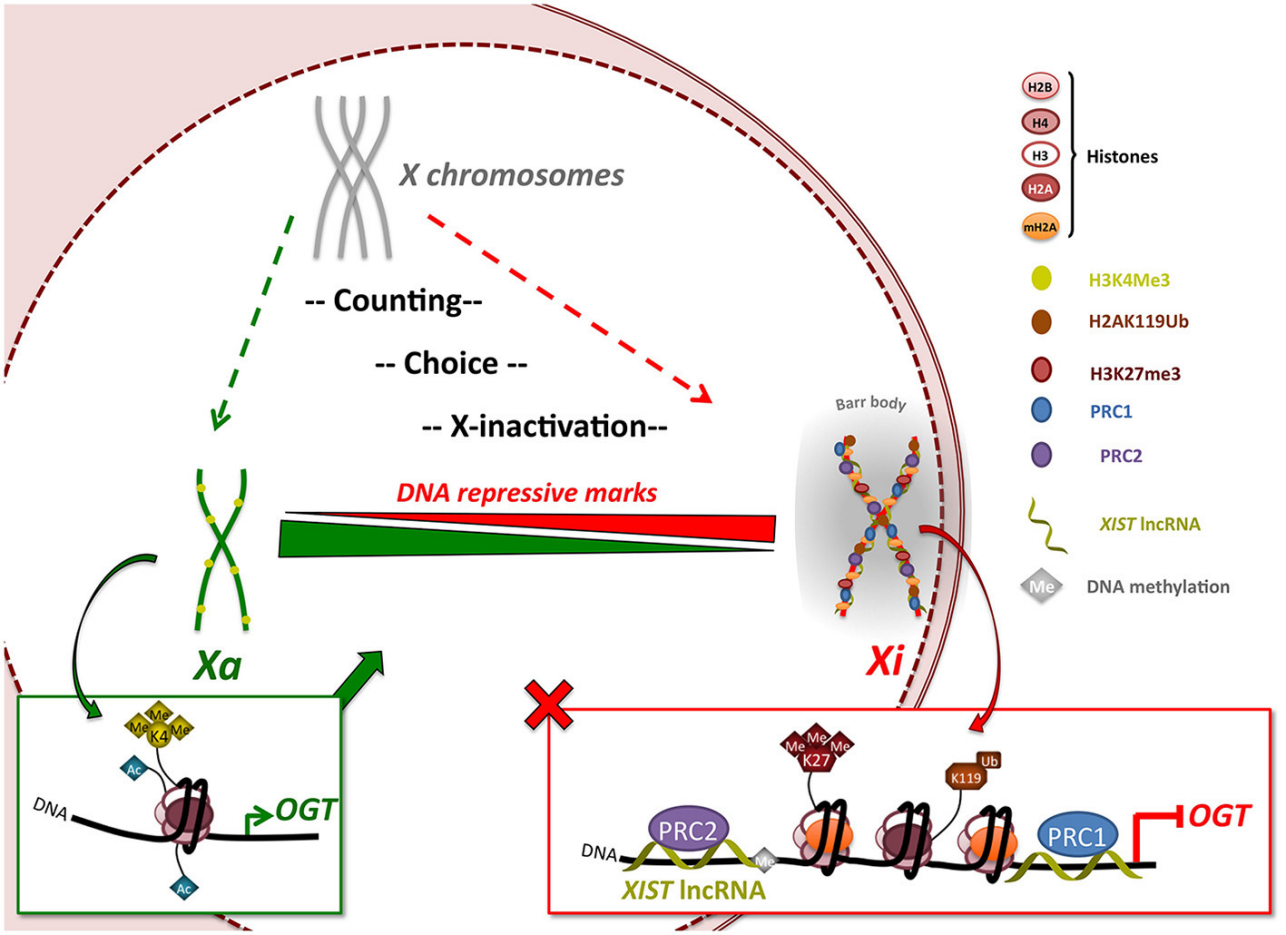
***OGT* expression is regulated by X-inactivation in female mammals**. The X-inactivation process begins with a counting/choice of which chromosome to inactivate. X-inactivation leads to silencing of almost the entire X-chromosome, including the *XIST*-proximal *OGT* gene, by recruitment of polycomb group proteins via *XIST* lncRNA and appearance of DNA repressive marks such as H3K27me3, H2AK119Ub and mH2A1. Once inactivation is completed, the inactivated X-chromosome (Xi) is re-localized in a highly compacted structure called the Barr body, from which OGT protein and *O*-GlcNAcylation are largely excluded indicating a highly transcriptionally silenced region. This process allows the dosage of OGT protein to be normalized in expression independent of the number of X-chromosomes. Escape from X-inactivation can lead to increases in *OGT* expression. *Xa: active X-chromosome*.

The presence of mammalian *OGT* on the X-chromosome has raised the question as to whether it is silenced or escapes X-inactivation. In fact, *OGT* is located close to the X-inactivation center (XIC) (Brown et al., 1991; Shafi et al., 2000; Hanover, 2001), suggesting a highly silenced location (Figure 4). Whereas previous studies have indicated that *Ogt* is subject to X-inactivation in mice (Lin et al., 2007), its transcriptional status remained largely unknown in humans. Because many more X-genes escape X-inactivation (15%) in humans than in mice (3%) (Carrel and Willard, 2005; Yang et al., 2010) it remained imperative to investigate *OGT* gene dosage in human cells. Here, we definitively show that indeed *OGT* is subject to X-inactivation in humans. Importantly, understanding regulation of *OGT* provides insights into human diseases in which aberrant *O*-GlcNAcylation has been correlated with disease progression.

In this study, we demonstrate that additional alleles of *OGT* are silenced in human female cells. We show that the *OGT* gene is subjected to X-inactivation in human fibroblasts to maintain near constant levels of OGT independent of X-chromosome number. As a consequence, *OGT* expression is equal in male, female or triple-X female cells. These three cell lines have 1, 2 or 3 X chromosomes, respectively, and only one should be active, with the remaining X-chromosomes inactivated. We demonstrated that the *OGT* locus co-immunoprecipitated with *XIST* lncRNA, mH2A1 and ubiquitinylated lysine 119 of histone H2A, key components of X-inactivation process and of the Barr body, suggesting that *OGT* is potentially subject to X-inactivation. Additionally, we have demonstrated that removal of DNA methylation triggers derepression of *OGT* in female and triple-X female cells. In the same manner, knock down of *XIST* lncRNA, increased OGT expression, further demonstrating X-inactivation-dependent repression of *OGT*. Moreover, we also showed that the *OGT* primary transcript does not co-localized with *XIST* lncRNA-coated chromosomes. Analysis of DNA encoding *OGT* indicated association with X-inactivation complex members, such as as mH2A1, and *XIST* lncRNA, Taken together, these findings show that *OGT* is subject to X-inactivation and raise many questions about whether *OGT* reactivation may be implicated in diseases that show a gender bias preferentially affecting females. We have suggested that *OGT* may be one of the X-linked genes serving as a driver of diseases involving chromosome imbalance (Abramowitz et al., 2014; Olivier-Van Stichelen et al., 2014).

In fact, aberrant methylation levels have been implicated in lupus development. Interestingly, this autoimmune disease has a higher prevalence in females and Klinefelter syndrome males (XXY), both of whom have 2 X-chromosomes, than in normal males. It has been suggested that demethylation of the silenced X-chromosome causes re-expression of many X-linked genes, including *OGT*, in these patients (Hewagama et al., 2013). These authors have posited that the increased OGT expression and *O*-GlcNAcylation level, may trigger or worsen lupus development (Hewagama et al., 2013). Moreover, deregulation of the X-inactivation process is also involved in human cancer development, often through methylation defects causing reactivation of silenced genes (Karpf and Jones, 2002; Liao et al., 2003; Pageau et al., 2007). Indeed, many cancers have been identified to have increased *O*-GlcNAc levels (Fardini et al., 2012). Moreover, multiple female-associated cancers, like breast and endometrial, have increased OGT and/or *O*-GlcNAc levels (Slawson et al., 2001; Gu et al., 2010; Krześlak et al., 2012; Champattanachai et al., 2013). Even if other non-gender specific cancers also have increased *O*-GlcNAcylation (Fardini et al., 2012), reactivation of Xi in female could potentially worsen or accelerate the process in the female patient.

In a second set of experiments, we endeavored to determine whether OGT and *O*-GlcNAcylation could be involved in the X-inactivation process itself. Indeed, *O*-GlcNAcylation has been found associated with chromatin preparations (Kelly and Hart, 1989), on transcription factors (Ozcan et al., 2010), on RNA polymerase II (Comer and Hart, 2001; Ranuncolo et al., 2012), and on histones H2A, H2B, H3, and H4 (Sakabe et al., 2010). Our lab has also shown that *O*-GlcNAcylation is particularly abundant on the promoter region in *C. elegans* (Love et al., 2010). Furthermore, OGT is also associated with TET (Ten-Elevent Translocation) proteins, responsible for demethylation of DNA and activation of transcription (Mariappa et al., 2013). Taken together, these studies implicate *O*-GlcNAcylation in chromatin remodeling and gene regulation, important features for X-inactivation.

In this study, we demonstrate that the Barr body, hosting the inactivated X-chromosome, is almost entirely devoid of *O*-GlcNAcylation. Moreover, OGT is also largely excluded from this perinuclear structure. During the X-inactivation process, Xi acquires silenced histone marks like trimethylated lysine 27 of histone 3, ubiquitinylated lysine 119 of histone 2A and mH2A1 (Mermoud et al., 1999; Plath et al., 2003; De Napoles et al., 2004). By this process, Xi is transformed to an ultra-condensed chromatin structure and we have demonstrated that it almost completely excludes *O*-GlcNAcylation and OGT. By definition, this structure also excludes the elongating form of RNA polymerase II (Phospho-S), although we show non-elongating RNA polymerase II in this region by pan RNA polymerase II antibody staining. As previously demonstrated, RNA polymerase II is *O*-GlcNAcylated on the C-terminal domain and *O*-GlcNAcylated RNA polymerase II is a transient state during the initiation complex formation (Ranuncolo et al., 2012). *O*-GlcNAcylation of RNA polymerase II is thought to occur in pre-initiation state before CTD kinases may act. In the case of a silenced Barr body, it is intriguing that of *O*-GlcNAcylation and RNA polymerase II do not colocalize in this completely silenced region of DNA. The absence of *O*-GlcNAcylation also corresponded with strong DNA inactivation marks, such as mH2A1, H3K27Me3 and H2AK119Ub. Consequently, the Barr bodies were neither enriched in *O*-GlcNAcylation nor OGT suggesting that the RNA polymerase II present there is not heavily *O*-GlcNAc modified in the Barr body. Furthermore, *O*-GlcNAcylation modulation does not effect the Xa/Xi distribution, confirming that X-inactivation and Barr body formation does not require *O*-GlcNAc cycling. This finding is consistent with a role for OGT and *O*-GlcNAcylation in transcription initiation, as phosphorylated isoforms of RNA polymerase II are also excluded from the Barr body.

The data presented herein support that random X-inactivation in adult female cells silences all but one of the *OGT* alleles leading to normalized gene expression (Figure 4). This process is distinct from the imprinted X-inactivation that occurs in the trophoblast where OGT sometimes escapes inactivation (Reviewed in Olivier-Van Stichelen et al., 2014). X-inactivation in the human female involves a multistep process involving an X-chromosome counting mechanism, choice of Xa/Xi and subsequent X-inactivation. The creation of a condensed structure isolates the Xi and the extra-OGT allele in a peri-nuclear area. Consequently, OGT levels are maintained independently of X-chromosome number. Intriguingly, OGT and *O*-GlcNAcylation are mostly excluded in the Barr body as detected by immunofluorescence and chromosome immunoprecipitation. This observation supports a role for OGT and *O*-GlcNAcylation in transcription initiation rather than silencing of DNA, although X-inactivation is known to require polycomb group complexes PRC2, which may associate with OGT (Myers et al., 2011; Chu et al., 2014). Nevertheless, *O*-GlcNAcylation may act upstream of Barr body formation to regulate the PRC2 complex. Thus, our findings suggest that while *O*-GlcNAc cycling may play a role in PRC2 function leading to X-inactivation, high levels of *O*-GlcNAc are not required to maintain the strong transcriptional repression necessary for silencing in the Xi/Barr body.

## Author contributions

Stéphanie Olivier-Van Stichelen designed and performed experiments, analyzed data and wrote the paper; John A. Hanover supervised the project, discussed the results and wrote portions of the manuscript.

### Conflict of interest statement

The authors declare that the research was conducted in the absence of any commercial or financial relationships that could be construed as a potential conflict of interest.

## Supplementary material

Figure S1 **Barr bodies exclude *O*-GlcNAcylated proteins and elongating RNA polymerase II (phospho S2/5). (A)** RNA-FISH specificity is confirmed by positive and negative control sets, using PPIB/RNA pol II mRNA and Bacterial mRNA probes, respectively. **(B)** An additional elongating RNA polymerase II (phospho S2/5) antibody confirms the exclusion of *O*-GlcNAcylation from Barr Bodies. **(C)** Fluorescent secondary antibodies alone show the specificity of primary antibodies used in this study. **(D)** Efficiency of Thiamet G and azaserine is demonstrated respectively by increased of decreased staining of *O*-GlcNAc antibody. Immunofluorescence pictures are representative of triplicate experiments.
