# Supplementary figures and images for: X-inactivation normalizes O-GlcNAc transferase levels and generates an O-GlcNAc-depleted Barr body
Source: Front Genet. 2014 Aug 4;5:256. doi: 10.3389/fgene.2014.00256 (PMC4120696; doi:10.3389/fgene.2014.00256)

## Slide 1
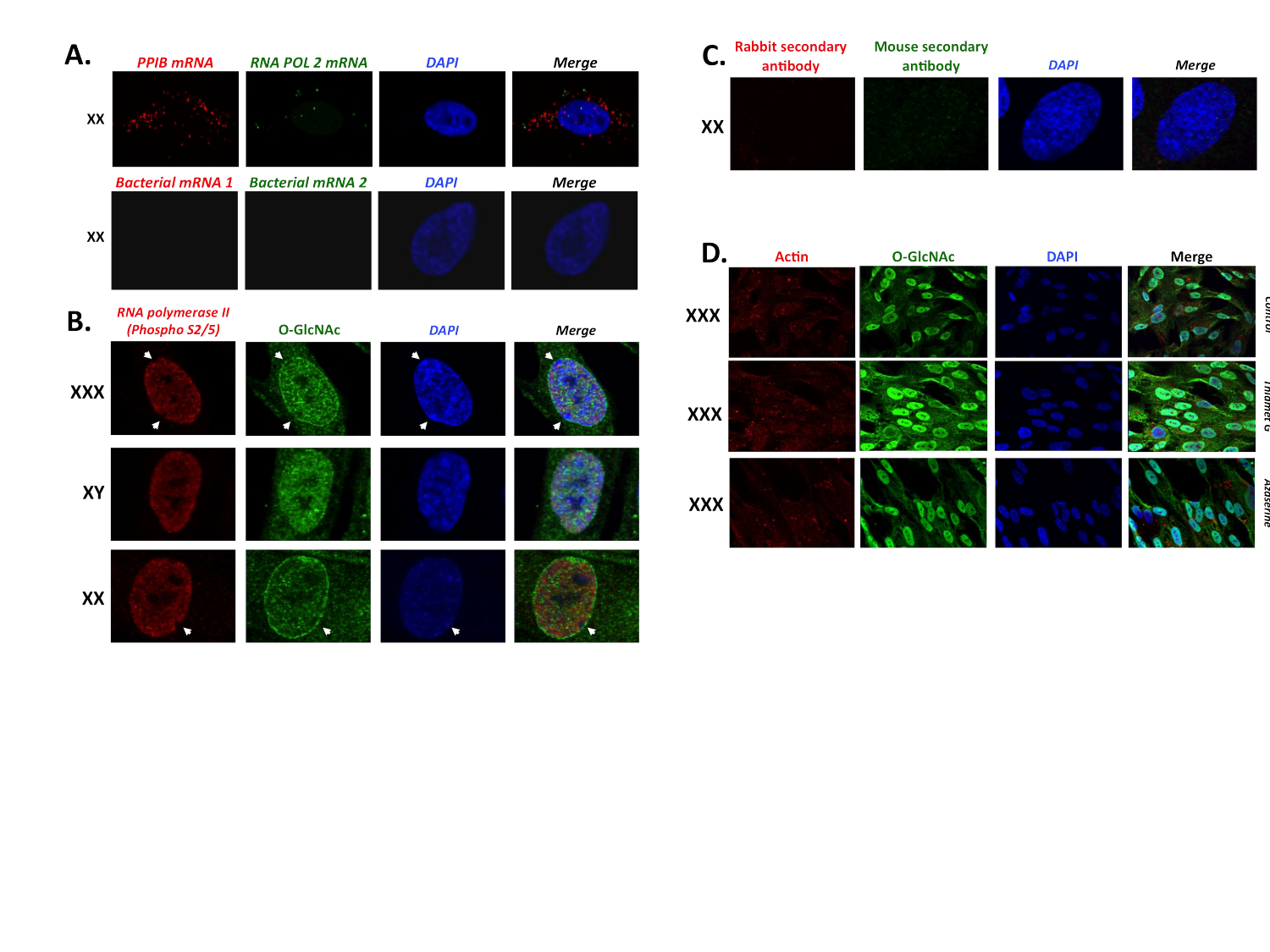

Supplement: Figure S1 — Barr bodies exclude O-GlcNAcylated proteins and elongating RNA polymerase II (phospho S2/5). (A) RNA-FISH specificity is confirmed by positive and negative control sets, using PPIB/RNA pol II mRNA and Bacterial mRNA probes, respectively. (B) An additional elongating RNA polymerase II (phospho S2/5) antibody confirms the exclusion of O-GlcNAcylation from Barr Bodies. (C) Fluorescent secondary antibodies alone show the specificity of primary antibodies used in this study. (D) Efficiency of Thiamet G and azaserine is demonstrated respectively by increased of decreased staining of O-GlcNAc antibody. Immunofluorescence pictures are representative of triplicate experiments. [file Presentation1.PPTX]
